# Clinical outcomes and treatment necessity in patients with toxin-negative *Clostridioides difficile* stool samples

**DOI:** 10.1186/s12941-024-00696-1

**Published:** 2024-04-25

**Authors:** Dae Hyeon Cho, Si-Ho Kim, Cheon Hoo Jeon, Hyoung Tae Kim, Kyoung-Jin Park, Junyoung Kim, Jiyeong Kwak, Byung Soo Kwan, Sungmin Kong, Jung Won Lee, Kwang Min Kim, Yu Mi Wi

**Affiliations:** 1 Department of Internal Medicine, Samsung Changwon Hospital, Sungkyunkwan University School of Medicine, Changwon, Korea; 2 Division of Infectious Diseases, Department of Internal Medicine, Samsung Changwon Hospital, Sungkyunkwan University School of Medicine, 50 Hapseong-dong, Masanhoewon-gu, Changwon-si, 51353 Gyeongsangnam-do Republic of Korea; 3 Department of Laboratory Medicine, Samsung Changwon Hospital, Sungkyunkwan University School of Medicine, Changwon, Republic of Korea

**Keywords:** *Clostridioides difficile*, Polymerase chain reaction, Enzyme immunoassay, Toxin EIA-positive CDI, Toxin EIA-negative CDI

## Abstract

**Purpose:**

The clinical significance of negative toxin enzyme immunoassays (EIA) for *Clostridioides difficile* infections (CDIs) is unclear. Our study aimed to investigate the significance of toxin EIA-negative in the diagnosis and prognosis of CDI.

**Methods:**

All stool specimens submitted for *C. difficile* toxin EIA testing were cultured to isolate *C. difficile*. In-house PCR for *tcdA*, *tcdB*, *cdtA*, and *cdtB* genes were performed using *C. difficile* isolates. Stool specimens were tested with *C. difficile* toxins A and B using EIA kit (RIDASCREEN *Clostridium difficile* toxin A/B, R-Biopharm AG, Darmstadt, Germany). Characteristics and subsequent CDI episodes of toxin EIA-negative and -positive patients were compared.

**Results:**

Among 190 *C. difficile* PCR-positive patients, 83 (43.7%) were toxin EIA-negative. Multivariate analysis revealed independent associations toxin EIA-negative results and shorter hospital stays (OR = 0.98, 95% CI 0.96–0.99, *p* = 0.013) and less high-risk antibiotic exposure in the preceding month (OR = 0.38, 95% CI 0.16–0.94, *p* = 0.035). Toxin EIA-negative patients displayed a significantly lower white blood cell count rate (11.0 vs. 35.4%, *p* < 0.001). Among the 54 patients who were toxin EIA-negative and did not receive CDI treatment, three (5.6%) were diagnosed with CDI after 7–21 days without complication.

**Conclusion:**

Our study demonstrates that toxin EIA-negative patients had milder laboratory findings and no complications, despite not receiving treatment. Prolonged hospitalisation and exposure to high-risk antibiotics could potentially serve as markers for the development of toxin EIA-positive CDI.

**Supplementary Information:**

The online version contains supplementary material available at 10.1186/s12941-024-00696-1.

## Introduction

Prompt and precise diagnosis of *Clostridioides difficile* infection (CDI) is crucial to initiate appropriate antibiotic therapy and control its transmission [1, 2]. Commonly used assays include *C. difficile* toxin enzyme immunoassays (EIAs) and toxin gene polymerase chain reaction (PCR) [1]. Toxin EIA is easy to use and rapidly provides results. However, there exists an underestimation of actual prevalence of CDI due to its poor sensitivity [3]. In contrast, toxin gene PCR is sensitive but cannot differentiate between active infections and asymptomatic carriage [4].

Previous studies have shown a robust correlation between toxin EIA-positive and CDI severity, as well as higher recurrence and/or increased mortality rates [4–8]. A toxin EIA-negative result implies asymptomatic colonization or mild CDI [4–8]. However, an intriguing subset of patients with CDI presents a diagnostic challenge, being *C. difficile* toxin gene PCR-positive but toxin EIA-negative. These patients pose a clinical conundrum as some may experience severe and complicated infections [9, 10]. This discrepancy raises questions about the clinical significance of toxin EIA-negative in CDI. Our study aimed to investigate the significance of toxin EIA-negative in the diagnosis and prognosis of CDI.

## Methods

We prospectively included all stool specimens submitted for *C. difficile* toxin EIA between July 2017 and June 2018 at a 760-bed hospital and retrospectively collected clinical data from the medical records. A total of 1,182 fresh stool specimens were analysed. Specimens were tested with *C. difficile* toxins A and B using EIA) kit (RIDASCREEN *Clostridium difficile* toxin A/B, R-Biopharm AG, Darmstadt, Germany) and cultivated as previously described on the day of specimen receipt [11]. DNA was extracted from the colonies grown on Brucella agar plates. *C. difficile* isolates were identified by 16s rRNA sequencing of the extracted DNA. To identify the toxin genes, PCR for *tcdA, tcdB, cdtA, cdtB*, and internal control gene (*tpi*) were performed as previously described [12, 13]. Patients were classified into two groups based on their test results: toxin EIA-positive and -negative. The definition of CDI encompassed patients with documented diarrhoea along with a positive result in either *C. difficile* EIA toxin assay or toxin gene PCR. Demographic information and clinical history were collected from all eligible patients. The progression of CDI over a 60-day was monitored through a thorough review of medical records. Laboratory results were obtained on the same day as the diarrhea diagnosis, and if those results were not available, results from within 1 day (either 1 day before or 1 day after) were used. Prior length of hospitalization encompassed the duration of the index admission before the manifestation of diarrheal symptoms. For patients transferred from external healthcare institutions, the hospitalization period at the referring facility was incorporated into the prior length of hospitalization. Prior antibiotic use was further classified according to the risk of contributing to the incidence or progression of CDI (high-, medium-, and low-risk antibiotics), as previously described [14].

Categorical data were presented as frequencies and percentages, while continuous variables were expressed as mean ± standard deviation or median and interquartile range, depending on their distribution. Normality was assessed using the Shapiro–Wilk test. Characteristics were compared utilizing appropriate statistical methods, such as the χ2 test, Fisher’s exact test, two-sample t-test, or Mann–Whitney U-test. A multivariate logistic regression model was used to determine the predictors of toxin EIA-positive for CDI. Variables with *p* < 0.10 in the univariable analysis were entered into the multivariate analysis. The adequacy of the final model was assessed using the Hosmer–Lemeshow statistic. Statistical Package for the Social Sciences for Windows (version 18.0; SPSS Inc., Chicago, IL, USA) was utilized for analyses.

## Results

During the study period, 190 patients exhibited positive for *C. difficile* by toxin gene PCR. Of these isolates, 83 (43.7%) were toxin EIA-negative and 107 (56.3%) were toxin EIA-positive. A comparison of baseline characteristics of toxin EIA-negative versus -positive patients (Table 1) revealed that a majority of the patients (60.5%) were elderly, aged 65 years or older, without a significant difference in age distribution between the toxin EIA-negative and EIA-positive groups. The gender ratio was balanced, with slightly more than half (51.1%) of the patients being male, and the proportion of males was comparable in both the negative and positive groups. Regarding underlying medical conditions, cerebrovascular disease was the most prevalent, affecting over one-third (37.4%) of the patients, followed by solid tumors (22.1%) and diabetes (20.5%). The prevalence of these chronic diseases did not differ significantly between the toxin EIA-negative and EIA-positive groups.

**Table 1:** Baseline characteristics of 190 patients with toxin EIA-negative stool samples or toxin EIA-positive for *Clostridiodes difficile*

	Total population (*n* = 190)	Toxin EIA - (*n* = 83)	Toxin EIA + (*n* = 107)	*p*-value
Age ≥ 65 years	115 (60.5)	47 (56.6)	68 (63.6)	0.333
Male sex	97 (51.1)	44 (53.0)	53 (49.5)	0.634
Hospital stay, days, median (IQR)	6.0 (1.0–20.0)	4.0 (0–14.5)	7.5 (1.0–25.3)	0.014
ICU at diagnosis	26 (13.7)	14 (16.9)	12 (11.2)	0.238
Category of infection				0.269
Community-onset	14 (7.4)	9 (10.8)	5 (4.7)	
Community-onset healthcare facility associated	56 (29.5)	24 (28.9)	32 (29.9)	
Hospital onset	120 (63.2)	50 (60.2)	70 (65.4)	
Underlying disease				
Diabetes	39 (20.5)	20 (24.1)	19 (17.8)	0.283
Cerebrovascular disease	71 (37.4)	27 (32.5)	44 (41.1)	0.225
Cardiovascular disease	41 (21.6)	20 (24.1)	21 (19.6)	0.458
Chronic lung disease	15 (7.9)	6 (7.2)	9 (8.4)	0.764
Liver cirrhosis	8 (4.2)	5 (6.0)	3 (2.8)	0.300
Chronic renal disease	16 (8.4)	9 (10.8)	7 (6.5)	0.290
Solid tumour	42 (22.1)	20 (24.1)	22 (20.6)	0.560
Solid organ transplantation	2 (1.1)	0 (0.0)	2 (1.9)	0.505
Charlson’s score, median (IQR)	2 (1–5)	3 (1–5)	2 (1–5)	0.503
Previous medical history within 1 month				
Operation	42 (22.1)	17 (20.5)	25 (23.4)	0.635
Immunosuppression	16 (8.4)	5 (6.0)	11 (10.3)	0.295
Antibiotic exposure (*n* = 182)				0.006
No exposure	24 (13.2)	16 (20.0)	8 (7.8)	
^c^Low-risk	9 (4.9)	5 (6.3)	4 (3.9)	
^b^Medium-risk	22 (12.1)	11 (13.8)	11 (10.8)	
^a^High-risk	127 (69.8)	48 (60.0)	79 (77.5)	
Gastrointestinal medication use at diagnosis				
Proton pump inhibitor	69 (36.9)	26 (32.1)	43 (40.6)	0.234
H2 receptor antagonist	62 (33.2)	27 (33.3)	35 (33.0)	0.964
Probiotics	31 (16.6)	12 (14.8)	19 (17.9)	0.571

EIA, enzyme immunoassay; IQR, interquartile range; ICU, intensive care unit; SD, Standard deviation; CRP, C-reactive protein; CDI, *C. difficile* infection

Data are n (%) unless otherwise stated

^a^High-risk antibiotics: carbapenem, 2nd-, 3rd-, or 4th-generation cephalosporin, fluoroquinolone, lincosamide, pivampicillin, or temocillin

^b^Medium-risk antibiotics: penicillin, penicillin combination, 1st-generation cephalosporin, macrolide, monobactam, or streptogramin

^c^Low-risk antibiotic: all other systemic antibiotics

A comparison of toxin EIA-negative versus -positive patients revealed a significantly shorter median prior hospital stay for toxin EIA-negative (4.0 vs. 7.5 d, *p* = 0.014). Patients who had a stool with toxin-negative test result were less likely to have received high-risk antibiotics in the preceding month (60.0 vs. 77.5%, *p* = 0.006). Multivariate analysis revealed shorter prior hospital stays (odds ratio [OR] = 0.98, 95% confidence interval [CI] 0.96–0.99, *p* = 0.013) and less high-risk antibiotic therapy in the preceding month (OR = 0.38, 95% CI 0.16–0.94, *p* = 0.035) as independent predictors of toxin EIA-negative patients (Table 2).

**Table 2:** Multivariable logistic regression analyses for independent predictors for negative toxin EIA for *Clostridiodes difficile*

	OR (95% CI)	*p*-value	Adjusted OR (95% CI)^a^	*p*-value
Hospital stay	0.97 (0.95–0.99)	0.007	0.98 (0.96–0.99)	0.013
Antibiotic exposure within 1 month				
No exposure	1			
^c^Low-risk	0.63 (0.13–2.94)	0.556		
^b^Medium-risk	0.50 (0.15–1.64)	0.254		
^a^High-risk	0.30 (0.12–0.76)	0.011	0.38 (0.16–0.94)	0.035

OR, odds ratio; CI, confidence interval

^a^ Variables with *p* < 0.1 in the univariate analyses are included in the subsequent multivariate regression model

Hosmer and Lemeshow test, χ2 = 6.911, *p* = 0.546

^a^High-risk antibiotics: carbapenem, 2nd-, 3rd-, or 4th-generation cephalosporin, fluoroquinolone, lincosamide, pivampicillin, or temocillin

^b^Medium-risk antibiotics: penicillin, penicillin combination, 1st-generation cephalosporin, macrolide, monobactam, or streptogramin

^c^Low-risk antibiotic: all other systemic antibiotics

Although most baseline clinical parameters were relatively indistinguishable between EIA- and EIA+, a significantly lower proportion in the toxin EIA-negative group exhibited a white blood cell count > 15,000 /µL compared to the toxin EIA-positive group, demonstrating a statistically significant difference (11.0 vs. 35.4%; *p* < 0.001). Although not statistically significant, trends in other laboratory parameters including C-reactive protein levels (median 38.6 vs. 57.5 mmol/L) and albumin levels (mean 3.4 vs. 3.1 g/dL) indicated potential associations with absence or presence of toxins in the stool sample. CDI treatment was initiated less frequently in the toxin-negative group compared to toxin-positive group (34.9 vs. 89.7%, *p* < 0.001), with no significant difference in mortality (8.5 vs. 7.6%, *p* = 0.819) or subsequent CDI episodes within 60 d (4.8 vs. 8.4%, *p* = 0.331) (Table 3). Among the 54 patients who were toxin EIA-negative and did not receive any CDI treatment, three (5.6%) were diagnosed with CDI without any complications.

**Table 3:** Comparison of clinical signs and outcomes of 190 patients with toxin EIA-negative stool samples or toxin EIA-positive for *Clostridiodes difficile*

Signs at diagnosis		Toxin EIA - (*n* = 83)	Toxin EIA + (*n* = 107)	
Body temperature > 38.0 °C	85 (44.7)	34 (41.0)	51 (47.7)	0.357
Shock	17 (9.2)	8 (10.1)	9 (8.6)	0.718
Ileus	9 (4.9)	3 (3.8)	6 (5.8)	0.734
Laboratory finding				
White blood cell count > 15,000 /µL	44 (24.9)	9 (11.0)	35 (35.4)	< 0.001
Acute kidney injury	12 (6.9)	6 (7.9)	6 (6.2)	0.661
Albumin level, g/dL, mean ± SD	3.2 (2.7–3.8)	3.4 (2.9–4.0)	3.1 (2.7–3.7)	0.057
C-reactive protein, mmol/L, median (IQR)	48.5 (13.2–95.4)	38.6 (7.9–85.5)	57.5 (19.7–106.5)	0.058
*CDI Treatments	125 (65.8)	29 (34.9)	96 (89.7)	< 0.001
Initial metronidazole	96 (50.5)	24 (28.9)	72 (67.3)	
Initial vancomycin	22 (11.6)	3 (3.6)	19 (17.8)	
Concomitant use of antibiotics during anti-CDI treatment	97 (51.1)	45 (54.2)	52 (48.6)	0.442
30 d mortality	15 (8.0)	7 (8.5)	8 (7.6)	0.819
30 d mortality among CDI treatment (*n* = 125)	10 (8.0)	3 (10.3)	7 (7.3)	0.696
CDI development within 60 d	13 (6.3)	4 (4.8)	9 (8.4)	0.331
CDI development within 60 d among CDI treatment (*n* = 125)	10 (8.0)	1 (3.4)	9 (9.4)	0.451

SD, standard deviation; IQR, interquartile range; CDI, *C. difficile* infection

Data are presented as n (%) unless otherwise stated. *Metronidazole and vancomycin

## Discussion

In the current study, 43.7% of PCR-positive patients lacked detectable toxin via toxin EIA. These PCR-positive but toxin EIA-negative patients were associated with a lower incidence of prolonged hospital stays and reduced exposure to high-risk antibiotics in the month preceding the testing, compared to the toxin EIA-positive patients. This aligns with prior evidence suggesting that CDI development might be preceded by hospitalisation and exposure to specific antibiotics [15]. The main risk factor for CDI is exposure to specific antibiotics, particularly clindamycin, cephalosporins, and fluoroquinolones [15]. Hospitalization itself is a significant risk factor for CDI due to the convergence of multiple risks, including antibiotic exposure, a spore-contaminated environment, inadequate hand hygiene by healthcare workers, and a highly susceptible elderly patient population [15]. Patients with toxin EIA-negative results were less likely to receive treatment compared to those with toxin EIA-positive results for *C. difficile*. Despite the lower treatment rate in the EIA-negative group, only a small number of patients (*n* = 3) were subsequently diagnosed with CDI upon repeat testing, which was analyzed as an indicator of persistent clinical suspicion. The interval between the initial toxin EIA-negative result and the subsequent toxin EIA-positive result for these three patients ranged from 7 to 21 days. Notably, none of these three patients who developed CDI after the initial negative testing experienced complications such as megacolon, fulminant colitis requiring colectomy, or intensive care unit admission related to CDI. This suggests a possible higher level of *C. difficile* colonization that did not warrant treatment within the toxin EIA-negative subgroup.

Among 190 PCR-positive isolates, 83 (43.7%) were toxin EIA-negative. This is consistent with earlier findings, where 55.3% (162/293) of PCR-positive patients were identified as toxin EIA-negative [4]. Guerrero et al. also reported 43 patients (32.6%) with negative EIA results for toxins among 132 individuals diagnosed with CDI by PCR [10]. These patients can be colonized with *C. difficile* along with an alternative cause of diarrhoea. Multiple studies have substantiated the frequent colonisation by *C. difficile* in hospitalised patients, with a considerable portion of nosocomial diarrhoea cases stemming from non-infectious origins [16, 17]. As PCR detects *C. difficile* toxin gene rather than stool toxins directly, CDI diagnoses based solely on PCR status may misclassify colonized patients as infected [4–6]. Furthermore, toxin EIA-negative patients could potentially represent mild or early stage infections, as clinical toxin tests may overlook toxins present at low concentrations, occasionally resulting in toxin EIA-negative patients who retest positive [4, 18, 19]. Prior research indicates that 8–9% of patients initially appear for toxin EIA positive for GDH or PCR later test positive in subsequent samples using toxin EIA [4, 18, 19]. Consistently, our investigation found that among the 54 patients who were initially toxin EIA-negative and did not receive treatment, only three (5.6%) were later diagnosed with CDI upon subsequent testing.

Our findings highlight longer prior hospital stays and recent high-risk antibiotic exposure as factors associated with toxin EIA positivity, suggesting their potential as markers of disease severity and contributing factors to CDI development. In addition, a significantly lower proportion of individuals in the toxin EIA-negative group exhibited a white blood cell count > 15,000 /µL compared to the toxin EIA-positive group. Although not statistically significant, trends in other laboratory parameters including C-reactive protein levels and albumin levels indicated potential associations with absence or presence of toxins in the stool sample. Previous studies [4–8] reported a positive association between EIA-positive stools and markers of severe disease, such as elevated white blood cell count and C-reactive protein levels [6]. Toxin EIA-positive stool samples were associated with increased white blood cell count and CRP, decreased renal function, and increased 30-day mortality compared to toxin EIA-negative stool samples [6]. Notably, a comprehensive prospective study revealed prolonged diarrhoea in patients positive for both toxin EIA and PCR, in contrast to those negative for toxin EIA but positive for PCR [4]. In a recent investigation, higher faecal *C. difficile* toxin levels were correlated with increased CDI severity and increased 30 d risk of mortality [7]. The extensive prospective study by Planche et al. [8] emphasised the clinical relevance of toxin EIA positivity, which is a pivotal diagnostic step in CDI, owing to its correlation with clinical outcomes.

On the other hand, our findings revealed that a considerable proportion (65.1%) of cases that were PCR-positive and toxin EIA-negative remained untreated, with only a small subset (5.6%) subsequently diagnosed with uncomplicated CDI. Congruently, the study by Yang et al. also identified a substantial proportion (65.1%) of PCR-positive and toxin EIA-negative cases that did not receive treatment, with merely a minor fraction (5.6%) later diagnosed with uncomplicated CDI [20]. However, the studies by Miller et al. [9] and Guerrero et al. [10] underscore the need for further investigations to delineate the appropriate management strategies for PCR-positive and toxin EIA-negative cases. Miller et al. identified predictors of CDI-attributable complications in PCR-positive and toxin EIA-negative patients, including severe baseline disease according to Infectious Diseases Society of America criteria, fulminant colitis at baseline, and fever exceeding 38.5 °C [9]. Guerrero et al. demonstrated clinical presentation similarities between EIA-negative and EIA-positive patients, with 21% of EIA-negative patients exhibiting severe CDI, including fatal fulminant CDI [10]. This underscores the necessity for further studies to delineate which subset of PCR-positive and toxin EIA-negative patients can resolve without treatment, requiring delayed treatment decisions, and establishing criteria for re-examination. Incorporating clinical and laboratory parameters, such as baseline disease severity, fulminant colitis, and fever, as suggested by Miller et al. [9], could aid in risk stratification and guide targeted treatment decisions for this subgroup.

Guidelines suggest incorporating a stool toxin assay within a multistep algorithm for diagnosing CDI [1, 21, 22]. This algorithm typically commences with either PCR or GDH assays, followed by further testing of positive specimens using toxin EIA [1]. This approach aims to curtail instances of overdiagnosis and subsequently mitigate the risk of overtreatment attributed to *C. difficile* colonisation. Withholding antibiotics appears safe for hospitalized patients exhibiting suspected CDI but testing toxin EIA-negative [23]. However, despite guidelines indicating that a negative toxin assay might signal colonisation, clinicians frequently opt for treatment in toxin gene PCR-positive and toxin EIA-negative cases in real-world setting [19]. A persistent percentage (between 70% and 78%) of patients with PCR-positive and toxin EIA-negative receive CDI treatment, even after implementing the two-step test procedure over a 4-year period [19]. The high negative predictive value of PCR might hold promise for infection prevention. However, its tendency toward overdiagnosis could lead to unnecessary treatment. Physician education is urgently needed and imperative, underscoring that molecular tests lack specificity for CDI, and that a positive PCR result does not necessarily mandate treatment, even in the presence of symptoms. Similarly, laboratories should recognize that rejecting formed stools does not preclude possibility of positive molecular tests indicating colonization rather than active infection.

The present study has some limitations. First, this single-center study possesses modest sample size. Validation of these findings requires a more extensive prospective study to ensure broader generalisability. Second, our study primarily observed strains 018, 002, and 017 among the circulating strains in our study population (Supplementary Table 1). The hypervirulent ribotype 027, which has been implicated in disease severity, excess morbidity, and elevated CDI recurrence rates, is not prevalent in Korean hospital settings [24]. Only one strain with 027 ribotype could be identified in our study. This limitation of strain diversity underscores the need for further explorations encompassing a wider array of strain types to better understand their potential impact on CDI dynamics and outcomes [25]. The third limitation is the absence of testing for other potential causative pathogens in stool samples that were PCR-positive and toxin EIA-negative. Clinical studies indicate that 12–32% of hospitalized patients develop diarrhea but ≤ 20% of cases are attributable to CDI [17]. While the presence of toxin genes was confirmed via PCR, the lack of toxin production may suggest that the diarrheal illness could have been attributable to other enteric pathogens. This limitation highlights the need for a more comprehensive diagnostic strategy when assessing diarrheal illnesses, particularly in cases where *C. difficile* is present but not producing toxins. As demonstrated in the study by Krutova et al., co-infections with other gastrointestinal pathogens, such as Campylobacter spp., rotavirus, and norovirus, can often occur in patients with suspected CDI [26]. In their evaluation of the mariPOC CDI and Gastro test, a multiplex antigen assay, the authors identified other causative agents of diarrhea in 4.53% of the samples initially requested for CDI testing. Notably, five samples showed concurrent positivity for *C. difficile* GDH and other pathogens (Campylobacter spp. or norovirus), highlighting the importance of comprehensive testing to detect potential co-infections. Therefore, further investigation of PCR-positive and toxin EIA-negative stool samples should involve testing for a broader range of enteric pathogens to identify potential co-infections or alternative etiologies for the diarrheal illness.

In conclusion, our study demonstrates that patients with toxin EIA-negative CDI can manifest milder laboratory findings, with no complications, despite not receiving treatment. Prolonged hospitalisation and exposure to high-risk antibiotics could potentially serve as markers for the development of toxin EIA-positive CDI. Subsequent extensive studies are imperative to ascertain the specific subset of PCR-positive and toxin EIA-negative patients who may naturally resolve without immediate treatment, necessitating delayed therapeutic decisions and warranting re-examination criteria.

### Electronic supplementary material

Below is the link to the electronic supplementary material.

Supplementary Material 1

## Data Availability

It is available from the corresponding author upon reasonable request.
