# Supplementary material for: Clinical outcomes and treatment necessity in patients with toxin-negative Clostridioides difficile stool samples
Source: Ann Clin Microbiol Antimicrob. 2024 Apr 25;23:35. doi: 10.1186/s12941-024-00696-1 (PMC11046793; doi:10.1186/s12941-024-00696-1)
Supplement: Supplementary file 1 — Supplementary Material 1 [file 12941_2024_696_MOESM1_ESM.docx]

**Supplement Table 1. Multivariable logistic regression analyses for independent predictors for negative toxin EIA for *Clostridiodes difficile***

|  | OR (95% CI) | *p*-value | Adjusted OR (95% CI)^a^ | *p*-value |
| --- | --- | --- | --- | --- |
| Hospital stay | 0.97 (0.95–0.99) | 0.007 | 0.98 (0.96–0.99) | 0.013 |
| Antibiotic exposure within 1 month |  |  |  |  |
| No exposure | 1 |  |  |  |
| ^c^Low-risk | 0.63 (0.13–2.94) | 0.556 |  |  |
| ^b^Medium-risk | 0.50 (0.15–1.64) | 0.254 |  |  |
| ^a^High-risk | 0.30 (0.12–0.76) | 0.011 | 0.38 (0.16–0.94) | 0.035 |

OR, odds ratio; CI, confidence interval

^a^ Variables with *p* <0.1 in the univariate analyses are included in the subsequent multivariate regression model.

Hosmer and Lemeshow test, χ2 = 6.911, *p* = 0.546

^a^High-risk antibiotics: carbapenem, 2nd-, 3rd-, or 4th-generation cephalosporin, fluoroquinolone, lincosamide, pivampicillin, or temocillin

^b^Medium-risk antibiotics: penicillin, penicillin combination, 1st-generation cephalosporin, macrolide, monobactam, or streptogramin

^c^Low-risk antibiotic: all other systemic antibiotics

**Supplemental Table 1. Ribotypes and antimicrobial resistance in 107 positive toxin EIA *C. difficile* isolates**

|  | | Resistant rate, n (%) | | | | | | | MIC (mg/L) | | |
| --- | --- | --- | --- | --- | --- | --- | --- | --- | --- | --- | --- |
| Antimicrobial agents | **Toxin gene**  **profile** | A+B+CDT-  (n = 90) | | | | A-B+CDT-  (n = 11) | | Total  (n = 107) | MIC_50_ | MIC_90_ | Range |
|  | **Ribotype** | 018  (n = 60) | 002  (n = 9) | 014/020  (n = 4) | C4  (n = 4) | 017  (n = 5) | B2  (n = 5) |  |  |  |  |
| CM |  | 58 (96.7) | 4 (44.4) | 3 (75.0) | 3 (75.0) | 4 (80.0) | 5 (100) | 83 (77.6) | ≥ 256 | ≥ 256 | 0.25-≥ 256 |
| MZ |  | 0 | 0 | 0 | 0 | 0 | 0 | 0 | 0.125 | 0.25 | 0.03-1 |
| TC |  | 0 | 0 | 1 (25.0) | 1 (25.0) | 4 (80.0) | 4 (80.0) | 12 (11.2) | 0.064 | 8 | 0.03-≥ 256 |
| TP |  |  |  |  |  |  |  | NA | 0.064 | 0.125 | 0.03-0.25 |
| VA |  | 0 | 0 | 0 | 0 | 0 | 0 | 0 | 0.5 | 1 | 0.12-2 |
| MX |  | 58 (96.7) | 8 (88.9) | 1 (25.0) | 0 | 4 (80.0) | 5 (100) | 73 (68.2) | ≥ 32 | ≥ 32 | 0.5-≥ 32 |
| RI |  |  |  |  |  |  |  | NA | 0.002 | ≥ 32 | 0.002-≥ 32 |

MIC, Minimum inhibitory concentration; R, resistant; CM, clindamycin; MIC, minimum inhibitory concentration; MX, moxifloxacin; MZ, metronidazole; NA, not applicable; R, resistant; RI, rifampicin; TC, tetracycline; TP, teicoplanin; VA, vancomycin.
